# Suppressing the Fluorescence Blinking of Single Quantum Dots Encased in N-type Semiconductor Nanoparticles

**DOI:** 10.1038/srep32662

**Published:** 2016-09-08

**Authors:** Bin Li, Guofeng Zhang, Zao Wang, Zhijie Li, Ruiyun Chen, Chengbing Qin, Yan Gao, Liantuan Xiao, Suotang Jia

**Affiliations:** 1 State Key Laboratory of Quantum Optics and Quantum Optics Devices, Institute of Laser Spectroscopy, Shanxi University, Taiyuan, 030006, China; 2 Collaborative Innovation Center of Extreme Optics, Shanxi University, Taiyuan, Shanxi, 030006, People’s Republic of China

## Abstract

N-type semiconductor indium tin oxide (ITO) nanoparticles are used to effectively suppress the fluorescence blinking of single near-infrared-emitting CdSeTe/ZnS core/shell quantum dots (QDs), where the ITO could block the electron transfer from excited QDs to trap states and facilitate more rapid regeneration of neutral QDs by back electron transfer. The average blinking rate of QDs is significantly reduced by more than an order of magnitude and the largest proportion of on-state is 98%, while the lifetime is not considerably reduced. Furthermore, an external electron transfer model is proposed to analyze the possible effect of radiative, nonradiative, and electron transfer pathways on fluorescence blinking. Theoretical analysis based on the model combined with measured results gives a quantitative insight into the blinking mechanism.

Colloidal quantum dots (QDs) are nanoscale single photon emitters with narrow, symmetric emission bands and high quantum efficiency, which have a wide range of applications such as molecular electronics^1^, photovoltaic devices^2^,^3^,^4^, photocatalysis^5^, and biomedical labelings^6^,^7^. For some specific applications, the near-infrared (NIR) emitting QDs, with different chemical composition and a bigger size, have attracted particular interest in biomedical labelings and photovoltaic materials^8^,^9^. There is a reduced absorption by biological tissues as well as the absence of autofluorescence from tissues in the NIR range. The NIR QDs are able to absorb NIR photons, as well as the visible photons, which can potentially improve the efficiency of solar cells. However, the QDs have intrinsic fluctuations of fluorescence intensity^10^,^11^, as called blinking, which are attributed to the photoinduced charging of QDs by electron transfer to trap states in QDs (or the surrounding matrix)^12^,^13^,^14^,^15^. The blinking behavior will reduce the photons generation rate^16^, cause difficulty in single particle tracking^17^, and degrade the performance of practical applications in photovoltaics and optoelectronics^4^. Hence, addressing and suppressing the undesirable blinking of existing core/shell QDs are extremely crucial.

Nowadays, many groups are enforcing their efforts on suppressing fluorescence blinking of QDs. The blinking was considered as random processes of ionization and neutralization under continuous laser excitation, such as Auger ionization and transient electron transfer from core to resonant energy states on or near the surface^18^. This rationale motivated researchers to investigate blinking suppression of QDs by perturbing the energy states of QDs, modifying Auger recombination rates, changing positive charged state back to the neutral state and so on. The typical techniques are to improve the route to synthesis the QDs^19^, surround the QDs with polymers^20^ or ligands^21^,^22^,^23^, encapsulate the core with higher band gap materials^24^,^25^,^26^,^27^ or thicker shell^28^,^29^,^30^, contact the QDs with noble metal nanoparticles/surfaces^31^,^32^ or semiconductor surfaces^13^,^15^, change the temperature^33^, reduce QDs’ contact with oxygen^34^, and change the excitation energy and intensity^35^,^36^. It has been shown that the fluorescence blinking of QDs could be suppressed very well, the percentage of fluorescence bright states could be up to 90%^21^,^37^ and the average blinking rate was reduced to one fifth of that on glass^13^. However, when QDs were spin-coated onto high-quality TiO_2_ semiconductor film, the charge carriers from photoactivated QDs can delocalize at greater extent by hopping among TiO_2_ particles. Thus, back electron transfer can be delayed, and then the duration of off-state events was increased and the lifetime was decreased^18^,^38^. The average lifetime values were reduced to 10% in some reports^13^,^27^. In addition, there is no feasible method reported for suppressing the fluorescence blinking of NIR QDs. Furthermore, there still lacks of the qualitative analysis about the blinking mechanism.

In this work, we apply N-type indium tin oxide (ITO) nanoparticles as semiconductor material to encase single NIR CdSeTe/ZnS core/shell QDs to suppress the fluorescence blinking. The ITO, with ~10 wt. % SnO_2_ doping, has a higher Fermi level than that of QDs, therefore the electrons in ITO will be transferred to QDs to fill in the trap states and then block the electron transfer from excited QDs to trap states. Furthermore, ITO is suitable for the applications in NIR QD-based optoelectronic devices due to its high transmission in the NIR region.

## Results

### Fluorescence radiation properties of single QDs in ITO

**Figure 1:**
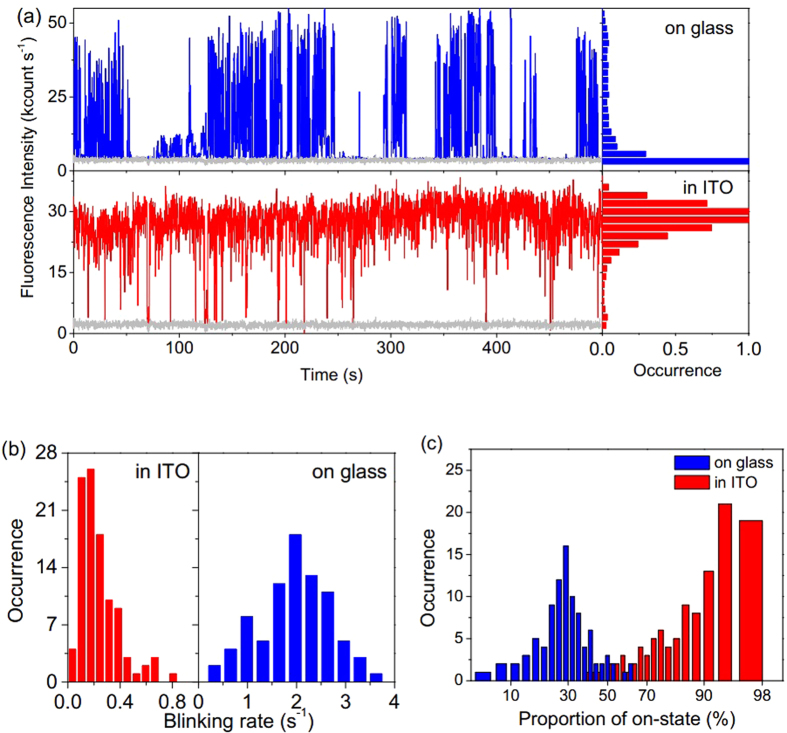
(**a**) Typical fluorescence intensity trajectories for the single QDs on glass coverslips and encased in ITO, respectively. The blue trajectory represents fluorescence intensity of single QD on glass coverslip and the red trajectory represents fluorescence intensity of single QD encased in ITO; the silver-gray trajectories represent background; the corresponding fluorescence intensity distribution is shown in the right panels. (**b**) Histograms of blinking rates for ~110 studied single QDs on glass coverslips and encased in ITO, respectively. (**c**) Histograms of proportion of on-state for ~110 studied single QDs on glass coverslips and encased in ITO, respectively.

The fluorescence intensity trajectories for single QDs on glass coverslips and encased in ITO were recorded by the confocal scanning fluorescence microscope system. Figure 1a shows two typical fluorescence intensity trajectories and corresponding fluorescence intensity histograms for single QDs on glass coverslips and encased in ITO, respectively. The trajectories were recorded with an integration time of 100 ms. It is found in the upper part of Fig. 1a that the fluorescence of single QDs on glass coverslips shows a quite strong blinking and the corresponding intensity histogram mainly lies on dark state. Compared with the results on glass coverslips, single QDs in ITO have less fluorescence blinking, and the corresponding intensity histogram mainly lies on bright state, as shown in the lower part of Fig. 1a.

In order to investigate the blinking activities of single QDs on glass coverslips and encased in ITO, we have calculated the blinking rate, the number of blinking events per second over 500 s long trajectories, for all measured single QDs. A blinking event is defined as a transition between on and off states^13^. The threshold fluorescence intensity, *I*_th_, is defined to separate the on and off states, *I*_th_ = *I*_av_ + 3*σ*, where *I*_av_ is the average fluorescence intensity of the background, and *σ* is its standard deviation. Figure 1b shows the histograms of fluorescence blinking rate for single QDs on glass coverslips and encased in ITO, respectively. The fluorescence blinking rate histograms were obtained from the fluorescence intensity trajectories for ~110 single QDs in the two cases. The peaks are at the blinking rates of 1.98 Hz (blue) and 0.17 Hz (red) for single QDs on glass coverslips and in ITO, respectively. Note that ITO significantly reduces the average blinking rate by more than an order of magnitude.

In addition, we have calculated the proportion of the number of occurrences of an on-state to the total number of samples with the integration time of 100 ms for single QDs on glass coverslips and encased in ITO, respectively. Histograms of the proportion of on-state are showed in Fig. 1c, the peaks of which are at 29% and 95% for single QDs on glass coverslips and encased in ITO, respectively. For a small percentage of the QDs in ITO, the proportion of on-state is down to 50% due to some occasional off states with a relatively long duration. The largest proportion of on-state of QDs reaches to 98%, which indicates that ITO can strongly suppress the fluorescence blinking of QDs.

### Normalized probability density distribution for single QDs

**Figure 2:**
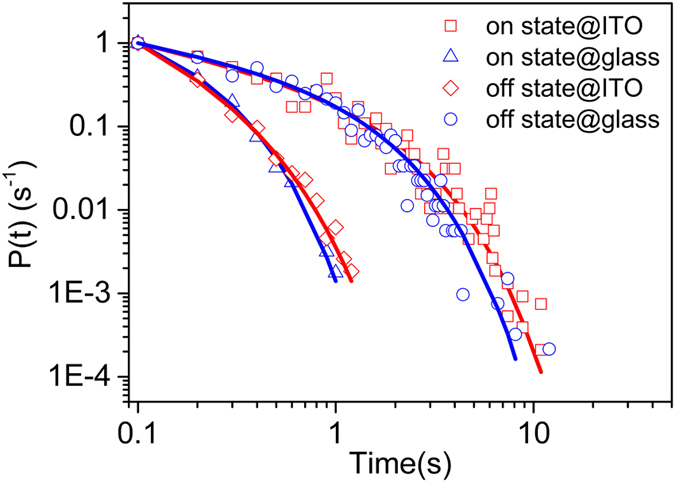
Normalized probability density of on-states (*P*_on_(*t*)) and off-states (*P*_off_(*t*)) for single QDs on glass coverslips and encased in ITO, respectively. The solid lines are best fits by a truncated power law. Fitting parameters for QDs on glass coverslips: *α*_on_ = 0.447, *α*_off_ = 0.435, 1/*μ*_on_ = 0.163, and 1/*μ*_off_ = 1.175; fitting parameters for QDs encased in ITO: *α*_on_ = 0.529, *α*_off_ = 0.965, 1/*μ*_on_ = 1.639, and 1/*μ*_off_ = 0.264.

The on and off states probability densities *P*_on_(*t*) and *P*_off_(*t*) of single QDs are used to compare the blinking activity of QDs on glass coverslips and encased in ITO, which have been calculated according to the method of Kuno *et al.*, 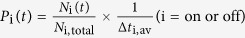
11. Where *N*_i_(*t*) is the statistics of on- or off-state events in duration time of *t*, *N*_i,total_ is the total number of on- or off- state events and 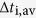 is the average of the time intervals to the preceding and following events. *P*_on_(*t*) and *P*_off_(*t*) of single QDs in the two cases show a power law distribution at short time but deviate from this distribution at long time tails, as shown in Fig. 2. These *P*_on_(*t*) and *P*_off_(*t*) distributions can be fitted by a truncated power law^10^,^39^,^40^: 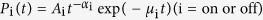, where *A* is the amplitude, *α* is the power law exponent, and *μ* is the saturation rate. In Fig. 2, the probability density of on-state at the duration time of 1s for single QDs in ITO is two orders of magnitude higher than that on glass coverslips. The fitting parameters for *α* and *μ* have been obtained by the fitting of ~110 single QDs on glass coverslips and encased in ITO respectively, as showed in Table 1. Single QDs encased in ITO have a larger 1/*μ*_on_ and a smaller 1/*μ*_off_ than that of QDs on glass coverslips, which suggests increased probability densities of on-state events and decreased probability densities of off-state events.

### Fluorescence lifetimes of single QDs encased in ITO

**Figure 3:**
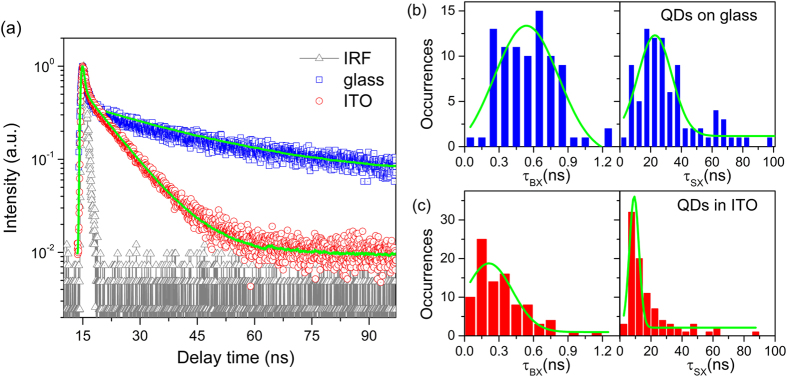
**(a)** Fluorescence decays and best biexponential fits for single QDs on glass coverslip and encased in ITO, respectively. IRF indicates the instrument response function of system. **(b,c)** Histograms of lifetimes for single-exciton states (*τ*_SX_) and biexciton states (*τ*_BX_) for single QDs on glass coverslips and that encased in ITO with Gaussian fitting (green curves), respectively.

To gain more insight into the emission dynamics, the fluorescence decay curves of single QDs on glass coverslips and encased in ITO were measured by using TAC&MCA method. The typical fluorescence decay curves are shown in Fig. 3a. The curve with blue open squares is the fluorescence decay of single QD on glass coverslips, and the curve with red open circles is the fluorescence decay of single QD encased in ITO. The curve of grey open triangles represents instrument respond function (IRF) of system with a full width at half maximum (FWHM) of about 750 ps. The decay curves can be fitted well by using a biexponential function with a longer lifetime component and a shorter one. The longer lifetime component can be assigned to the relaxation of single-exciton (SX) states^41^,^42^, while the shorter one can be assigned to the relaxation of biexciton (BX) states^42^,^43^,^44^. The BX states can undergo either radiative decay or nonradiative Auger relaxation (NR-AR) pathways^45^. In general, the rate of NR-AR is much larger than that of radiative decay^42^. The relaxation lifetimes for both SX and BX states could be extracted by fitting decay curves, 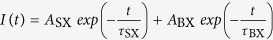, where *τ*_SX_ and *τ*_BX_ are fluorescence lifetimes of SX and BX states, respectively^13^,^42^, *A*_SX_ and *A*_BX_ are corresponding amplitudes. Actually, the measured decay curve *F*(*t*) was a scatter convolution of its response to the excitation light flash *G*(*t*), one need to deconvolve the decay curve with the equitation, 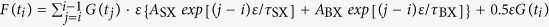, to determine the real lifetimes^46^.

In Fig. 3a, the fitting parameters for the single QD’s fluorescence decay curve on glass coverslips are *τ*_SX_ = 28.8 ns, *w*_SX_ = 87.4%, *τ*_BX_ = 0.61 ns and *w*_BX_ = 12.6%. *w*_SX_ and *w*_BX_ are the amplitude weights defined as 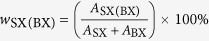. From the amplitude-weighted average lifetime of single QDs obtained by 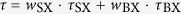, we can get the amplitude-weighted average lifetime 
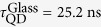
. The result corresponds to the ref. 13 where the single QDs were also prepared onto glass coverslips. For the QDs encased in ITO, the fitting parameters for the single QD’s fluorescence decay curve are 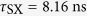, *w*_SX_ = 83.8%, 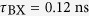, *w*_BX_ = 16.2% and then the amplitude-weighted average lifetime is 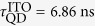. The amplitude-weighted average lifetimes for 120 single QDs on glass coverslips and encased in ITO are 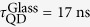 and 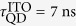, respectively. The histograms of lifetime values *τ*_SX_ and *τ*_BX_ for 120 single QDs on glass coverslips and encased in ITO are shown in Fig. 3b,c. The histograms were fitted by Gaussian functions, and the lifetime values at the Gauss peaks for single QDs on glass coverslips are about 22.7 ns (*τ*_SX_) and 0.54 ns (*τ*_BX_) with the FWHMs of 28.5 ns (*τ*_SX_) and 0.64 ns (*τ*_BX_), respectively. They reduce to 9.2 ns (*τ*_SX_) and 0.22 ns (*τ*_BX_) with the FWHMs of 7.2 ns (*τ*_SX_) and 0.49 ns (*τ*_BX_) respectively in the case encased in ITO. The lifetime values for single-exciton states and biexciton states are reduced to ~41% and ~34% that of QDs on glass coverslips, respectively. The reduced lifetimes are attributed to extra nonradiative processes introduced by ITO, and the extra nonradiative processes include NR-AR, electron and hole transfer processes^13^,^42^.

Fluorescence quantum yields of the CdSeTe/ZnS core/shell QDs on glass coverslips is ~70%^47^, the radiative decay rate is 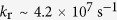, and the nonradiative decay rate is 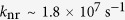. For QDs in ITO, *k*_r_ and *k*_nr_ can be estimated by the average fluorescence intensity QDs encased in ITO, as shown in Table 2. Combining with 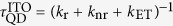, the electron transfer rate (*k*_ET_) from excited QDs to ITO can be calculated, as shown in Table 2.

## Discussion

The N-type semiconductor ITO nanoparticles have a higher Fermi level than that of QDs and the Fermi level of ITO is located above its conduction level (the calculations of Fermi levels are presented in the Supplementary Information)^13^. Therefore, when contacted, there is a driving force for the electron transfer from ITO to QDs until their Fermi levels come in balance and the excess electrons in QDs will fill in the trap states below the balanced Fermi levels to suppress the fluorescence blinking. The trap states are located in the shell near the interface^48^, and their energy levels positions locate between conduction band and Fermi level of QDs^13^,^49^, and the density distribution of trap states is a Gaussian blow the conduction band edge^49^.

**Figure 4:**
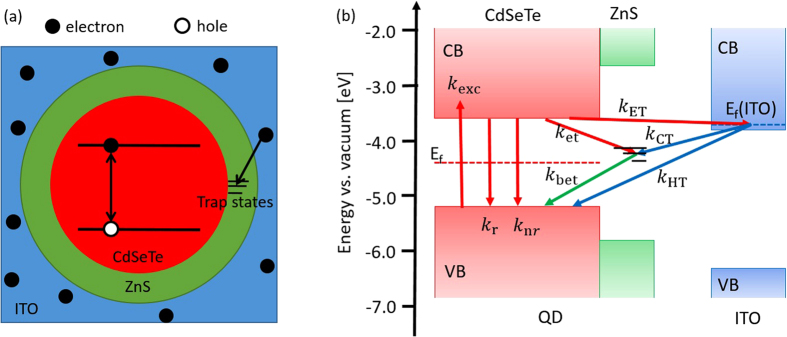
**(a)** Cutaway view of single QD encased in ITO and removing the trap states in QD’s shell by electron transfer. **(b)** Schematic of the excitation-relaxation cycle of single QD and possible charge transfer pathways between QD and ITO. CB and VB are the conduction band and valence band, respectively; *E*_*f*_ is the Fermi level; *k*_exc_ is the excitation rate, *k*_r_ is the radiative decay rate, *k*_nr_ is the nonradiative decay rate; *k*_et_ indicates the electron transfer from excited state to trap state; *k*_bet_ is the electron transfer rate from the trap state to the ground state of QD; *k*_ET_ is the rate of electron transfer from excited QD to ITO; *k*_CT_ indicates the electron transfer from ITO to trap state; *k*_HT_ indicates the electron transfer from ITO to the ground state (hole) of QD.

Based on the experimental results, we propose an external electron transfer model for the fluorescence blinking suppression of QDs by modifying surface-trap-filling model^50^, the diffusive coordinate model^51^,^52^, and the interfacial electron transfer model as we reported previously^53^, as shown in Fig. 4. The schematic of the excitation-relaxation cycle of QD and possible electron transfer pathways between QD and ITO nanoparticles is described by the following set of kinetic equations


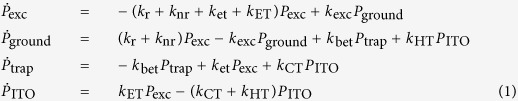


where *P*_exc_, *P*_ground_, *P*_trap_, and *P*_ITO_ are the population probability of the excited state, the ground state, the trap state, and the electronic *E*_*f*_ energy (Fermi level) of ITO, respectively. *k*_exc_, *k*_r_, *k*_nr_, *k*_et_, *k*_bet_, *k*_ET_, *k*_CT_, and *k*_HT_ represent for excitation rate, radiative decay rate, nonradiative decay rate, electron transfer rate from excited state to trap state, electron transfer rate from the trap state to the ground state of QD, electron transfer rate from excited QD to the ITO, electron transfer from the ITO to trap state, and electron transfer from ITO to the ground state (hole) of QD, respectively. The populations satisfy the normalization condition *P*_exc_ + *P*_ground_ + *P*_trap_ + *P*_ITO_ = 1. The main parameters we will discuss in this paper are the probability of on- and off-state which can be described by *P*_on_ = *P*_exc_ + *P*_ground_ + *P*_ITO_ and *P*_off_ = *P*_trap_. The probabilities of QD remaining in the on and off states must approach to zero when their duration approaches to infinity. Equations for these functions can be derived with the help of Equations (1) as follows.

### On states

In order to find equations for the distribution function for on-state we should omit term *k*_bet_*P*_trap_ in the second equation of (1). This term defines transitions to on-state. Since *P*_ITO_ is a constant due to the abundant electrons in ITO, we may set 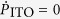, that is 
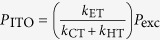
. For QDs in contact with *n*-doped ITO, electrons in ITO will be no longer transferred to QDs when their Fermi levels are equilibrated, so the *k*_CT_ = 0. The excitation rate is expressed as 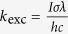
10, where *I* and *λ* are the intensity and the wavelength of the excited light, and *σ* is the absorption cross section. The *λ* = 635 nm, and *I* = 400 W/cm^2^ in our experiment corresponds to an excitation rate 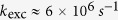 for QDs on glass, using 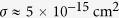 for the absorption cross section of the CdSeTe/ZnS QDs^54^. For QDs encased in ITO, the *k*_exc_ is estimated to be ~9.6 × 10^6^ s^−1^ according to the ref. 42. The radiative decay rate *k*_r_ is the inverse of the radiative lifetime *τ*_*r*_ (~20 ns at room temperature), so *k*_exc_/*k*_r_ ≪ 1. The population probability *P*_exc_ of the excited state is always much less than unity with 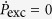. In this approximation we find from the first equation of (1) the relation 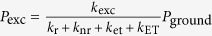. The neighboring ITO nanoparticles provide fast nonradiative decay pathways to facilitate the nonradiative exciton recombination, as *k*_ET_ ≈ *k*_HT_. Then the probability of on-state can be calculated as


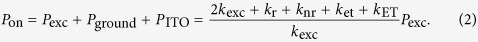


The sum of Equations (1) yields the following equation:


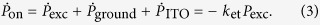


To combine Equations (2) and (3), we can get


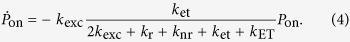


The derivative of the survival probability of the on-state is a distribution of the on-time, 
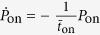
, where


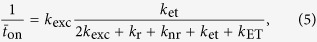


and the 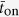 is the average on-time of QDs.

### Off states

In order to find equations for the distribution function for off states we should omit term *k*_et_*P*_exc_ in the third equation of (1). This term defines transitions to non-fluorescent states. After that we arrive at the equation 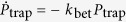, that is 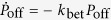. The rate of electron transfer (*k*_bet_) is simply the inverse of the average off-time,


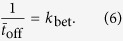


For QDs on glass coverslips, there are no electron transfers between QDs and ITO nanoparticles. We can get the following equations about 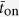 and 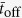,


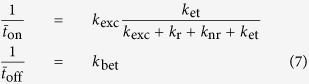


The values of 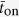 and 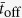 can be got by the integral of the probability densities of on-state and off-state, 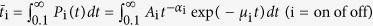, with the fitting parameters in Table 1. The values of 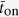 and 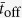 are shown in Table 2. Using Equations (5, 6, 7), we can calculate the *k*_et_ and the *k*_bet_, as shown in Table 2. The calculated results show that the ITO nanoparticles not only reduce the electron transfer rate from excited QD to trap states but also can accelerate the electron transfer rate from the trap states to the ground state of QD. *k*_et_ is reduced by ~54% and *k*_bet_ is increased by a factor of ~2.2.

## Conclusions

We have shown that the fluorescence blinking activity of NIR QDs can be significantly suppressed by encasing them in the N-type semiconductor ITO nanoparticles. Since the ITO has a higher Fermi level than that of QDs, the electrons in ITO would be transferred to QDs to fill in the trap states and then block the electron transfer from excited QDs to the trap states. The fluorescence blinking has been largely suppressed while the lifetime has not been considerably reduced. The external electron transfer model has been used to analyze the possible effect of the additional electron transfer pathways. The quantitative insight into the blinking mechanism based on the electron transfer between QDs and semiconductor materials presents prerequisite for developing QD-based optoelectronic devices.

## Methods

### Sample preparation

The NIR CdSeTe/ZnS core/shell QDs (Qdot^®^800ITKTM Organic Quantum Dots) were ordered from Thermo Fisher Scientific. Their maximum fluorescence emission wavelength is at 800 nm. The ITO (dispersion, <100 nm particle size (DLS), 30 wt. % in isopropanol, composition: In_2_O_3_ 90%, SnO_2_ 10%) was purified with a filter with pore size of 0.45 *μ*m. And then centrifuged it at 3000 rpm for 3 minutes, then we wiped out the supernatant, and the precipitate was dispersed in toluene with a concentration of ~15 wt. %. At last, QDs solution in toluene was added to the dispersion and formed a mixture with QDs concentration of ~10^−9^ mol/L. The mixture was spin coated onto a cleaned glass coverslip at a rotational speed of 3000 rpm to form an ITO film encased single QDs. The samples were placed in vacuum at 315 K for 3 hours to remove the residual solvent. We also prepared the contrast sample with only single QDs on glass coverslips as a control experiment. The glass coverslips (25 mm × 25 mm) were purchased from Ted Pella.

### Experimental setup

Confocal scanning fluorescence microscope system was employed to measure the fluorescence intensity and lifetime of single QDs^53^,^55^. The system was equipped with a picosecond pulsed diode laser (PDL800-D PicoQuant) with a central wavelength of 635 nm, output pulse width of 55 ps, and repetition frequency of 80 MHz. The laser light was led to an inversion microscope (Nikon ECLIPSE TE2000-U) through a single mode polarization maintain fiber. A *λ*/2 and a *λ*/4 wave-plate were used to change the linearly polarized laser into circular polarization light. An oil immersion objective (Nikon, 100×, 1.3 NA) was used to focus laser light onto the sample and collect fluorescence simultaneously. The fluorescence, passing through a dichroic mirror (BrightLine, Semrock), an emission filter (BrightLine, Semrock), and a notch filter (BrightLine, Semrock), was focused into a 100 μm pinhole for spatial filtering to reject out-of-focus photons. Finally, the fluorescence was collected by a single photon detector (PerkinElmer, SPCM-AQR-15). A piezo-scan stage (Tritor 200/20 SG) with an active x-y-z feedback loop mounted on the inversion microscope was used to scan the sample over the focused excitation spot. A time-to-amplitude converter (TAC, ORTEC) and a multi-channel analyzer (MCA, ORTEC) were used to measure the fluorescence decay curves to obtain fluorescence lifetime of QDs.

## Additional Information

**How to cite this article**: Li, B. *et al.* Suppressing the Fluorescence Blinking of Single Quantum Dots Encased in N-type Semiconductor Nanoparticles. *Sci. Rep.* **6**, 32662; doi: 10.1038/srep32662 (2016).

## Supplementary Material

Supplementary Information

## Figures and Tables

**Table 1:** Fitting parameters for normalized probability density of on-states (*P*_on_(*t*)) and off-states (*P*_off_(*t*)) for ~110 single QDs on glass coverslips and in ITO, respectively.

	*α*_*o*n_	1/*μ*_on_	*α*_off_	1/*μ*_off_
QDs (on Glass)	0.485 ± 0.187	0.319 ± 0.182	0.632 ± 0.274	0.913 ± 0.325
QDs (in ITO)	0.568 ± 0.163	2.583 ± 0.739	1.062 ± 0.573	0.221 ± 0.138

**Table 2:** Calculated parameters from single QDs on glass coverslips and in ITO.

	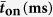 a	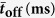 b	*k*_exc_(s^−1^)^c^	*k*_r_(s^−1^)^d^	*k*_nr_(s^−1^)^e^	*k*_et_(s^−1^)^f^	*k*_bet_(s^−1^)^g^	*k*_ET_(s^−1^)^h^
QD (on Glass)	188 ± 21	314 ± 30	~6 × 10^6^	~4.2 × 10^7^	~1.8 × 10^7^	58.5 ± 5.9	3.2 ± 0.3	~
QD (in ITO)	631 ± 90	94 ± 10	~9.6 × 10^6^	~2.5 × 10^7^	~3.5 × 10^7^	26.8 ± 3.3	10.1 ± 0.5	~8.3 × 10^7^

^a^Average on-time of single QDs on glass coverslips and in ITO.

^b^Average off-time of single QDs on glass coverslips and in ITO.

^c^Calculated excitation rate.

^d^Radiative decay rate.

^e^Nonradiative decay rate.

^f^Electron transfer rate from excited QD to trap states.

^g^Electron transfer rate from the trap states to the ground state of QD.

^h^Electron transfer rate from excited QD to ITO.
