# Supplementary material for: Suppressing the Fluorescence Blinking of Single Quantum Dots Encased in N-type Semiconductor Nanoparticles
Source: Sci Rep. 2016 Sep 8;6:32662. doi: 10.1038/srep32662 (PMC5015025; doi:10.1038/srep32662)
Supplement: Supplementary Information [file srep32662-s1.pdf]

Suppressing the Fluorescence Blinking of Single Quantum Dots Encased in N-type Semiconductor Nanoparticles

Bin Li[1,2], Guofeng Zhang[1,2], Zao Wang[1,2], Zhijie Li[1,2], Ruiyun Chen[1,2], Chengbing Qin[1,2], Yan Gao[1,2], Liantuan Xiao[1,2], Suotang Jia[1,2]

[1]State Key Laboratory of Quantum Optics and Quantum Optics Devices, Institute of Laser Spectroscopy, Shanxi University, Taiyuan, 030006, China.

[2]Collaborative Innovation Center of Extreme Optics, Shanxi University, Taiyuan, Shanxi, 030006, People's Republic of China.

Supplementary Information (SI)

Calculations of Fermi levels for ITO nanoparticles and quantum dots (QDs)

For the ITO (In_2O_3 with ~10% SnO_2 doping), the Fermi level of the ITO can be calculated by[1, 2]

$$E_f = E_i + kTln\left(\frac{N}{N_i}\right), \quad (1)$$

where E_i is the Fermi energy of intrinsic semiconductor (In_2O_3), and is assumed to be at the middle of the band gap for In_2O_3; N and N_i are conduction band electron density of the doped semiconductor (ITO) and intrinsic semiconductor (In_2O_3), respectively; k is the Bolzmann constant, T is the temperature.

The conduction band electron density of In_2O_3 can be calculated by[2, 3]

$$N_i(T) = 2.5\left(\frac{m_c m_v}{m_0^2}\right)^{3/4}\left(\frac{T}{300\text{ K}}\right)^{3/2} exp\left(-\frac{E_g}{2kT}\right) 10^{19}\text{ cm}^{-3}, \quad (2)$$

where E_g is the band gap energy of the ultra-pure In_2O_3 powder with a value of 2.5 eV[4], m_c and m_v are the effective mass of the conduction band electron and valence band hole, respectively, and m_0 is the mass of free electrons. Taking m_c=0.3m_0, m_v=0.6m_0 [5], we calculate N_i to be $\sim 4.95 \times 10^{-3}\ \mathrm{cm}^{-3}$ at 298 K. Taking $E_i = -5.07$ eV, $N = \sim 2.2 \times 10^{21}\ \mathrm{cm}^{-3}$ [6], the Fermi level of the ITO can be estimated to be -3.72eV.

For CdSeTe/ZnS core/shell QDs with the maximum fluorescence emission wavelength is at 800 nm, the conduction and valance band energies of CdSeTe are -3.62 eV and -5.17 eV, respectively[7]. Thus the Fermi level of the QDs can be estimated to be -4.40eV.

Therefore, the N-type semiconductor ITO nanoparticles have a higher Fermi level than that of the QDs.

SUPPLEMENTAL REFERENCES

1. Hunter, L. P. *Introduction to semiconductor phenomena and devices* (ed. Addison-Wesley) (Reading, MA, 1996).
2. Jin, S.; Song, N.; Lian, T. Suppressed blinking dynamics of single QDs on ITO. *ASC Nano* **4**, 1545-1552 (2010).
3. Ashcroft, N. W.; Mermin, N. D. *Solid state physics* (Thomson Learning: New York, 1976).
4. Wilson, E.; John, K.; Michael, G. Light-induced oxygen generation in aqueous dispersion of indium oxide (In_2O_3) particles and determination of their conduction

band position. *Chem. Phys. Lett.* **110,** 648–650 (1984).

5. Hamberg, I.; Granqvist, C. G. Evaporated Sn-doped In_2O_3 films: Basic optical properties and applications to energy-efficient windows. *J. Appl. Phys.* **60,** 123–159 (1986).
6. Brewer, S. H.; Franzen, S. Calculation of the electronic and optical properties of indium tin oxide by density functional theory. *Chem. Phys.* **300,** 285–293 (2004).
7. Pan, Z. X. et al. Near infrared absorption of $CdSe_xTe_{1-x}$ alloyed quantum dot sensitized solar cells with more than 6% efficiency and high stability. *ACS nano* **7**, 5215-5222 (2013)
